# Structural validity and test-retest reliability of the Patient Reported Inventory of Self-Management of Chronic Conditions (PRISM-CC) in a Swedish population of seventy-year-olds with long-term health conditions

**DOI:** 10.1186/s41687-025-00892-3

**Published:** 2025-05-28

**Authors:** Ingrid Olsson, George Kephart, Tanya Packer, Sabine Björk, Ulf Isaksson, Yu-Ting Chen, Anna Nordström, Åsa Audulv

**Affiliations:** 1 Department of Nursing, Umeå University, Biologihuset, Hus C, Plan 3, Umeå, 901 87 Sweden; 2 Department of Community Health and Epidemiology, Dalhousie University, Halifax, Canada; 3 School of Health Administration, Dalhousie University, Halifax, Canada; 4 School of Occupational Therapy, Dalhousie University, Halifax, Canada; 5 Department of Public Health and Clinical Medicine, Section of Sustainable Health, Umeå University, Umeå, Sweden; 6 Artic Centre, Umeå University, Umeå, Sweden; 7 Department of Occupational Therapy, College of Medicine, National Cheng Kung University, Tainan, Taiwan; 8 Department of Medical Sciences, Rehabilitation Medicine, Uppsala University, Uppsala University Hospital, Uppsala, Sweden; 9 School of Sport Sciences, UiT The Arctic University of Norway, Tromsø, Norway

**Keywords:** Patient reported outcome measurement, Self-management, Psychometrics, Long-term health conditions, Multimorbidity, Older adults

## Abstract

**Background:**

Self-management is internationally recognized as important to maintain independence, quality of life and to minimize the risk of poor health outcomes, especially among persons with multi-morbidity. Self-management can be especially challenging for older adults, who have higher rates of multi-morbidity and experience diverse impacts of long-term health conditions on everyday life. Good measures of self-management are currently lacking. The Patient Reported Inventory of Self-Management of Chronic Conditions (PRISM-CC) is a new, generic, multidimensional measure of self-perceived ease or difficulty with self-management, that overcomes many of the limitations of existing measures.

**Objectives:**

To test the structural validity and test-retest reliability of the Swedish version of the PRISM-CC among seventy-year-olds with long-term health conditions.

**Methods:**

Translation of PRISM-CC items into Swedish followed the Patient-Reported Outcome (PRO) Consortium process. Survey data (n = 516 Swedish seventy-year-olds with ≥1 long-term health condition) was used to assess structural validity of the 36-item PRISM-CC using multidimensional item response theory (IRT) models. Test-retest reliability was assessed on a subsample of 58 individuals using intra-class correlation coefficient (ICC) and Bland-Altman Plots.

**Results:**

The Swedish PRISM-CC demonstrated good internal consistency with Cronbach’s alpha >0.8 for all domains, and good fit to a graded response IRT model (RMSEA 0.034, SRMSR 0.050, CFI 0.952 and TLI 0.945). All 36 items had standardized loadings >0.7. ICC showed moderate to good test-retest reliability for all seven domains. The Bland-Altman plots showed minimal bias and good test-retest agreement for all domains.

**Conclusion:**

The Swedish PRISM-CC showed good structural validity and test-retest reliability in this sample of relatively healthy seventy-year-olds with long-term health condition(s). Further validation in a population with more severe health issues is needed.

**Supplementary information:**

The online version contains supplementary material available at 10.1186/s41687-025-00892-3.

## Background

With the ageing of the population, the prevalence of people living with long-term health conditions and multi-morbidity is increasing [1, 2]. This results in higher healthcare utilization and costs [3, 4] and often leads to poor health, reduced functional capacity [4] and diminished quality of life [5].

Self-management and self-management support are widely recognized as essential components of chronic disease management [6, 7] and have been shown to improve quality of life and reduce healthcare utilization [8]. For older adults, who, in addition to having a higher prevalence of multi-morbidity and/or associated disability [9], often experience disruptive life transitions such as retirement, loss of family and friends and changes in housing and economic situations [10], self-management can be particularly challenging [11]. Adequate self-management support can help older adults remain independent, benefiting both older individuals and society as a whole [12].

Self-management is described as the daily “tasks that individuals must undertake to live well with one or more chronic conditions” [12]. Research shows that self-management is multi-dimensional, including interrelated aspects of managing daily life [8, 13, 14]. These are captured by the Taxonomy of Everyday Self-management Strategies (TEDSS), which categorizes them into seven essential, interrelated domains delineating the work people do to manage the everyday consequences of long-term health conditions. The seven TEDSS domains are divided into five goal-oriented domains (Internal, Social Interaction, Activities, Healthy Behaviours and Disease Controlling) and two support-oriented domains (Process and Resource). Even though each domain is distinct, all domains are related [14, 15].

Research on self-management support interventions has grown substantially in the last decades [8]; however, outcome measures to evaluate the effectiveness of these interventions can be improved. Today, a variety of outcomes are used which makes self-management interventions difficult to compare [16, 17]. Among existing self-management measures, many are unidimensional, providing a single total score [18, 19]. Furthermore, many self-management measures are disease specific; yet self-management interventions are often used across different diagnostic groups, and many people need to manage multi-morbidity [20, 21]. The lack of self-management measures designed for multi-morbidity is particularly troublesome in primary and community care, where patients can have various and/or multiple conditions [22, 23]. The Health Education Impact Questionnaire (heiQ) is one exception. It is a generic measure developed to evaluate self-management interventions [24, 25] that partly covers six of the seven TEDSS domains (all except Activity). However, like other measures, it does not assess the full range of the self-management domains described in the TEDSS [20]. Measures that identify different dimensions of self-management are especially important to guide tailored self-management support for patients and to evaluate which aspects of interventions are effective.

The Patient Reported Inventory of Self-Management of Chronic Conditions (PRISM-CC) is a new instrument that overcomes the limitations of many existing measures. The PRISM-CC is a generic, multi-dimensional instrument of self-perceived ease or difficulty with self-management experienced by adults with one or more long-term health conditions [26, 27]. Measuring perceived difficulty across different domains helps differentiate individual needs for self-management support, which are likely to vary due to disease trajectory and severity, heterogeneity across conditions, life contexts, existing support, and abilities [15, 28]. The PRISM-CC is based on the TEDSS framework [14] and was developed in a Canadian-Swedish collaboration following the COnsensus-based Standards for the selection of health Measurement INstruments (COSMIN) guidelines [29]. The English version of the PRISM-CC has shown excellent internal consistency and construct validity [27]. Therefore, the aim of this study was to assess the structural validity and test-retest reliability of the concurrently developed Swedish version of the PRISM-CC among seventy-year-olds with various long-term health conditions.

## Methods

### Design

Development of the PRISM-CC adheres to the Patient Reported Outcomes Measurement Information System (PROMIS®) Instrument Development and Validation Scientific Standard v2.0 [30] and the COSMIN guidelines [29].

### Participants and data collection

Participants for this cross-sectional survey were recruited through the Healthy Aging Initiative (HAI) study [31], an ongoing study in Umeå, a municipality with approximately 130,000 residents in northern Sweden. The HAI study is population based and invites every community-dwelling adult to enrol in the study the year they turn 70 years of age. In this study we invited individuals who enrolled in the HAI in 2018–2019. Ethical approval for the HAI study was granted by the Swedish Ethical Review Authority and the Regional Ethics Review Board in Umeå in 2007 (Dnr 2012-85-32M- and dnr 07-031M), and a complementary ethical application for this study was approved in 2020 (Dnr 2020-02387).

Data were collected between May 2021 and February 2022, through a survey sent to 1117 previous HAI-participants. At the time for this study, they were 72–73 years old (Supplementary File 1). Participants were informed of the study by letter and gave informed consent before completing the survey, either online or by paper-and-pencil. The HAI enrols people with and without long-term health conditions. Therefore, all HAI study participants were invited to participate and having one or more long-term health condition(s) was stated as inclusion criteria. For test-retest purposes, a sub-sample of 100 participants were sent the PRISM-CC survey a second time. Those who answered the survey within three weeks of the first survey were included in the test-retest analysis.

### PRISM-CC development

**Fig. 1:**
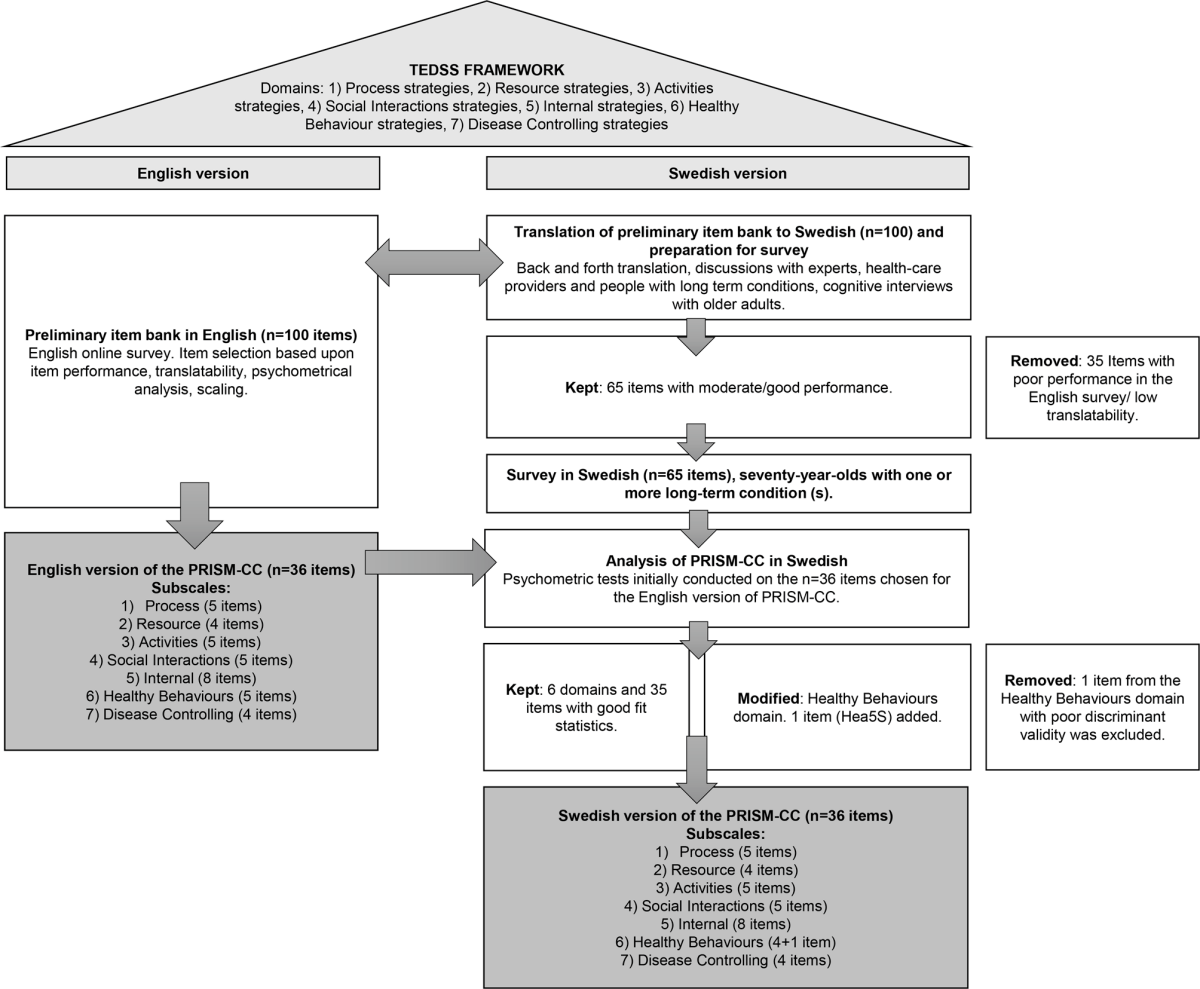
Development process of the English and Swedish versions of the PRISM-CC

The interconnected development of the English and Swedish versions of the PRISM-CC, is shown in Fig. 1. Development and testing of the English version has previously been reported [26, 27] and is here shortly summarised.

Based on the seven domains of the TEDSS framework, a preliminary item bank (n = 100) was constructed. Items were then tested and scaled in a step-by-step process using data from an online English survey. The final English version of the PRISM-CC consists of 36 items, with four to eight items per domain [27]. At the same time item selection and scaling of the English version of PRISM-CC was underway, the preliminary item bank (n = 100) was translated into Swedish. Information regarding translatability was used as one of the criteria, along with psychometric and conceptual analysis, for item selection in the English version. Thirty-five of the worst-performing items, which proved to have poor translatability, poor item-rest correlations and/or weak factor loadings in the English version, were not included in the Swedish survey. The Swedish survey thus included translations of 65 items, of which 36 were ultimately selected for the English version.

When responding to the potential PRISM-CC items, participants were encouraged to think about their health during a typical week. All items were formulated as statements, with a six-option response scale. The most common response scale assessed difficulty: “Unable to do”, “With extreme difficulty”, “With much difficulty”, “With little difficulty”, “Easily”, and “Very easily”. When this was not applicable, two other response scales were used: 1) “Never” to “Always” or 2) “Strongly disagree” to “Strongly agree”. Item responses were all scored 0 to 5, with higher scores indicating higher perceived self-management success. Each item also included a “not applicable” (NA) response option.

In addition to PRISM-CC items, the survey collected sociodemographic information (e.g., sex, marital status, highest education level, living arrangement and financial status), number and type of self-reported long-term health conditions and their perceived impact on everyday life. The item measuring impact on everyday life read: “How do you feel that your illness(es) affects your life?”: “not at all”, “a little bit”, “moderately”, “quite a bit”, “extremely”, or “I don´t know”.

### Translation process

The translation of PRISM-CC items into Swedish was guided by the Patient-Reported Outcome (PRO) Consortium translations process [32] recommended by PROMIS®. First, forward translation was conducted by a professional, native-speaking Swedish translator fluent in English. The interprofessional Canadian-Swedish research team then discussed the translation with the translator, Swedish experts in self-management, healthcare providers and older adults. Second, back translation was conducted by a professional, native-speaking English translator fluent in Swedish with no previous knowledge of the PRISM-CC items. The research team evaluated the forward-and-back translation to assess semantic equivalence and identify problematic items. Finally, cognitive interviews were performed via Zoom™ [33] with three participants that matched the target population. Minor amendments were made throughout the translation process to ensure that the items were understandable in Swedish, captured the essence of the items and were conceptually congruent with the domains. For example, for item 3 in the Disease Controlling domain – “I know which symptoms I need to act upon” – “act upon” was changed to “do something about” since “act upon” was perceived as being more formal and not commonly used in the Swedish language.

### Statistical methods

Analyses focused on assessing the Swedish version of the PRISM-CC based on translations of the 36 items selected for the English version. If indicated, alternative items from the translated Swedish item bank were assessed as potential alternatives to weakly performing items.

### Handling of missing data

Missing data resulted from both item non-response and from NA responses. Item non-response was uncommon (<2% for all items), but 11 items had NA response of >10% (Table 1). It was assumed that these two types of missing data were not missing completely at random and resulted from different processes. Although all subjects reported at least one self-reported chronic health condition, it was hypothesized that NA responses resulted from subjects’ perception that their condition(s) had no impact or relevance to these items. Fisher exact test and correlations confirmed hypotheses that 1) participants who reported their condition(s) had no impact on life would have a higher number of NA responses compared to participants who reported that their condition(s) had an impact on life, and 2) participants with one disease would have a higher number of NA responses compared to participants with comorbidities (Supplementary File 2). On the other hand, item non-response may have resulted from not understanding or having missed an item.

**Table 1:** Domain definitions, items included in the Swedish version of PRISM-CC and descriptive statistics (*n* = 516)

Domain, item and Swedish translation		Response Categories (Difficulty < - > Success)						N/A*	Missing
		0	1	2	3	4	5		
		*n* (%)	*n* (%)	*n* (%)	*n* (%)	*n* (%)	*n* (%)	*n* (%)	*n* (%)
**Resource**	**Self-perceived success in seeking, pursuing and/or managing needed formal or informal supports and resources.**								
Res1	When I have appointments with my healthcare providers, I tell them what I want or need.	2 (0.4)	4 (0.8)	7 (1.4)	32 (6.2)	191 (37.0)	213 (41.3)	62 (12.0)	5 (1.0)
	*När jag träffar mina vårdgivare berättar jag vad jag vill eller vad jag behöver.*								
Res2	I talk to my healthcare provider(s) about my condition(s).	4 (0.8)	5 (1.0)	4 (0.8)	24 (4.7)	192 (37.2)	181 (35.1)	100 (19.4)	6 (1.2)
	*Jag talar med mina vårdgivare om min(a) sjukdomar.*								
Res3	I arrange appointments with my health care provider(s).	6 (1.2)	1 (0.2)	7 (1.4)	26 (5.0)	189 (36.6)	230 (44.6)	52 (10.1)	5 (1.0)
	*Jag bokar in besök hos mina vårdgivare.*								
Res4	When I need to, I find people to help me understand information I receive about my condition(s).	4 (0.8)	2 (0.4)	2 (0.4)	21 (4.1)	167 (32.4)	151 (29.3)	161 (31.2)	8 (1.6)
	*När jag behöver det hittar jag människor som kan hjälpa mig att förstå information jag får om min(a) sjukdomar.*								
**Process**	**Self-perceived success in seeking information, being aware of choices and making good decisions.**								
Pro1	I identify what information I can trust.	8 (1.6)	5 (1.0)	2 (0.4)	54 (10.5)	216 (41.9)	151 (29.3)	75 (14.5)	5 (1.0)
	*Jag tar reda på vilken slags information jag kan lita på.*								
Pro2	I make informed decisions.	1 (0.2)	1 (0.2)	6 (1.2)	52 (10.1)	259 (50.2)	158 (30.6)	34 (6.6)	5 (1.0)
	*Jag fattar välgrundade beslut.*								
Pro3	I think about the consequences of different decisions.	…	3 (0.6)	7 (1.4)	49 (9.5)	237 (45.9)	173 (33.5)	38 (7.4)	9 (1.7)
	*Jag tänker på konsekvenserna av olika beslut.*								
Pro4	I try different things to find out what works best for me.	3 (0.6)	2 (0.4)	3 (0.6)	54 (10.5)	233 (45.2)	140 (27.1)	75 (14.5)	6 (1.2)
	*Jag provar olika saker för att ta reda på vad som fungerar bäst för mig.*								
Pro5	I keep myself updated with new information related to my health conditions.	3 (0.6)	5 (1.0)	9 (1.7)	59 (11.4)	218 (42.3)	173 (33.5)	47 (9.1)	2 (0.4)
	*Jag håller mig uppdaterad med aktuell information om min(a) sjukdomar.*								
**Internal**	**Self-perceived success in creating inner calm by preventing and managing stress, negative emotions, and internal distress.**								
Int1	I set realistic expectations for myself.	4 (0.8)	2 (0.4)	8 (1.6)	91 (17.6)	257 (49.8)	128 (24.8)	23 (4.5)	3 (0.6)
	*Jag sätter realistiska förväntningar på mig själv.*								
Int2	I accept the things I cannot change.	3 (0.6)	7 (1.4)	14 (2.7)	132 (25.6)	229 (44.4)	113 (21.9)	16 (3.1)	2 (0.4)
	*Jag accepterar saker jag inte kan förändra.*								
Int3	I manage my emotions and reactions.	1 (0.2)	6 (1.2)	9 (1.7)	124 (24.0)	247 (47.9)	122 (23.6)	5 (1.0)	2 (0.4)
	*Jag hanterar mina känslor och reaktioner.*								
Int4	I have and use ways to recover after a bad day.	5 (1.0)	4 (0.8)	7 (1.4)	91 (17.6)	242 (46.9)	125 (24.2)	41 (8.0)	1 (0.2)
	*Jag har och använder mig av olika sätt att återhämta mig efter en dålig dag.*								
Int5	I deal with frustration caused by my health situation.	2 (0.4)	4 (0.8)	12 (2.3)	135 (26.2)	202 (39.2)	82 (15.9)	76 (14.7)	3 (0.6)
	*Jag hanterar frustration som orsakas av min hälsosituation.*								
Int6	I manage my stress.	1 (0.2)	11 (2.1)	15 (2.9)	143 (27.7)	208 (40.3)	101 (19.6)	32 (6.2)	5 (1.0)
	*Jag hanterar min stress.*								
Int7	I focus on the positives.	…	7 (1.4)	10 (1.9)	106 (20.5)	241 (46.7)	142 (27.5)	5 (1.0)	5 (1.0)
	*Jag fokuserar på det positiva.*								
Int8	I forgive myself when I make a mistake.	2 (0.4)	12 (2.3)	22 (4.3)	143 (27.7)	228 (44.2)	90 (17.4)	16 (3.1)	3 (0.6)
	*Jag förlåter mig själv när jag gör misstag.*								
**Activity**	**Self-perceived success in participating in everyday activities (leisure activities, work activities, household chores).**								
Act1	I organize things in my home to make my life easier.	…	2 (0.4)	7 (1.4)	32 (6.2)	188 (36.4)	226 (43.8)	56 (10.9)	5 (1.0)
	*Jag organiserar saker i mitt hem för att underlätta mitt liv.*								
Act2	I plan ahead before going somewhere to be sure I can manage my health condition(s).	2 (0.4)	2 (0.4)	6 (1.2)	33 (6.4)	178 (34.5)	192 (37.2)	99 (19.2)	4 (0.8)
	*Jag planerar i förväg innan jag ska åka någonstans för att vara säker på att jag kan hantera min(a) sjukdomar.*								
Act3	I plan my time so I can get things done.	2 (0.4)	1 (0.2)	6 (1.2)	41 (8.0)	223 (43.2)	215 (41.7)	23 (4.5)	5 (1.0)
	*Jag planerar min tid så att jag kan få saker gjorda.*								
Act4	I manage my health condition(s) so that I can do things I enjoy.	2 (0.4)	4 (0.8)	9 (1.7)	68 (13.2)	194 (37.6)	203 (39.3)	29 (5.6)	7 (1.4)
	*Jag tar hand om min(a) sjukdomar så att jag kan göra saker som ger mig glädje.*								
Act5	I make time to do things I enjoy.	5 (1.0)	3 (0.6)	4 (0.8)	51 (9.9)	242 (46.9)	205 (39.7)	4 (0.8)	2 (0.4)
	*Jag avsätter tid för att göra saker som glädjer mig.*								
**Social Interaction**	**Self-perceived success in disclosing health issues, managing social interactions and relationships.**								
Soc1	I prioritize social interactions that I enjoy.	…	2 (0.4)	6 (1.2)	38 (7.4)	245 (47.5)	204 (39.5)	16 (3.1)	5 (1.0)
	*Jag prioriterar socialt umgänge som glädjer mig.*								
Soc2	I can explain my symptoms so family and friends can understand them.	3 (0.6)	6 (1.2)	3 (0.6)	57 (11.1)	230 (44.6)	182 (35.3)	31 (6.0)	4 (0.8)
	*Jag kan förklara mina symptom så att familj och vänner kan förstå dem.*								
Soc3	I clearly express my needs to others.	10 (1.9)	11 (2.1)	18 (3.5)	149 (28.9)	191 (37.0)	73 (14.2)	59 (11.4)	5 (1.0)
	*Jag uttrycker tydligt mina behov för andra.*								
Soc4	I devote time and attention to those who are dear to me.	1 (0.2)	1 (0.2)	2 (0.4)	33 (6.4)	250 (48.5)	216 (41.9)	9 (1.7)	4 (0.8)
	*Jag lägger tid och uppmärksamhet på människor som jag bryr mig om.*								
Soc5	When problems with my health arise, I stay in touch with people who are important to me.	3 (0.6)	1 (0.2)	4 (0.8)	46 (8.9)	233 (45.2)	168 (32.6)	56 (10.9)	5 (1.0)
	*När problem med min hälsa uppstår håller jag kontakten med människor som är viktiga för mig.*								
**Healthy Behaviour**	**Self-perceived success maintaining a healthy lifestyle in order to enhance health and limit the risk of lifestyle related illness**								
Hea1	I maintain healthy lifestyle behaviours that I know are important for my health.	5 (1.0)	1 (0.2)	10 (1.9)	73 (14.2)	249 (48.3)	169 (32.8)	5 (1.0)	4 (0.8)
	*Jag upprätthåller hälsosamma levnadsvanor som jag vet är viktiga för min hälsa.*								
Hea2	I make healthy food choices.	1 (0.2)	4 (0.8)	13 (2.5)	69 (13.4)	260 (50.4)	153 (29.7)	12 (2.3)	4 (0.8)
	*Jag väljer att äta nyttigt.*								
Hea3	I find ways to train my brain to keep mentally fit.	2 (0.4)	3 (0.6)	6 (1.2)	43 (8.3)	247 (47.9)	201 (39.0)	11 (2.1)	3 (0.6)
	*Jag hittar sätt att träna hjärnan för att hålla den igång.*								
Hea4	I create healthy sleeping habits.	6 (1.2)	12 (2.3)	30 (5.8)	93 (18.0)	231 (44.8)	136 (26.4)	5 (1.0)	3 (0.6)
	*Jag skapar hälsosamma sömnvanor.*								
Hea5S**	I maintain healthy behaviours even when I have a lot to do.	4 (0.8)	3 (0.6)	13 (2.5)	103 (20.0)	261 (50.6)	94 (18.2)	30 (5.8)	8 (1.6)
	*Jag upprätthåller hälsosamma vanor även när jag har mycket att göra.*								
**Disease Controlling**	**Self-perceived success in managing health conditions including managing medications and treatments, monitoring symptoms and limiting complications.**								
Dis1	When problems with my health arise, I understand what to do to manage my condition(s).	…	1 (0.2)	3 (0.6)	54 (10.5)	118 (22.9)	308 (59.7)	25 (4.8)	7 (1.4)
	*När jag får problem med min hälsa förstår jag vad jag kan göra för att ta hand om min(a) sjukdomar.*								
Dis2	I know what to do if I experience side-effects or other problems with my treatment or medication.	1 (0.2)	1 (0.2)	6 (1.2)	60 (11.6)	131 (25.4)	262 (50.8)	48 (9.3)	7 (1.4)
	*Jag vet vad jag ska göra om jag upplever biverkningar eller andra problem till följd av min behandling eller mina mediciner.*								
Dis3	I know which symptoms I need to act upon.	…	1 (0.2)	6 (1.2)	71 (13.8)	143 (27.7)	257 (49.8)	32 (6.2)	6 (1.2)
	*Jag vet vilka symptom som jag behöver göra något åt.*								
Dis4	I know what to do when my symptoms get worse.	2 (0.4)	…	4 (0.8)	64 (12.4)	134 (26.0)	278 (53.9)	27 (5.2)	7 (1.4)
	*Jag vet vad jag ska göra när mina symptom förvärras.*								

^*^N/A: not applicable

^**^Item exchanged in the Swedish version. Original item from English version: “I create time in my day to be active (walk to work, do housework, yard work or other daily activities”

Data from individuals with more than 50% NA responses or missing values (n = 14) were removed. Subsequently, chained multiple imputation with ordinal logistic regression in Stata 17 [34] was used to impute remaining item non-response and NA responses. As predictors, the imputation procedure employed all items from the same domain, together with respondents’ gender (male, female), level of education (incomplete elementary school, elementary school, high school, graduate degree) and the perceived impact of their health conditions on daily life (extremely, quite a bit, moderately, a little bit, not at all) [35, 36]. The imputation process was done in two distinct stages, for each of the two types of missing data. In the first stage, non-responses were imputed, and in the second stage, NA responses were imputed. The imputed values for NA responses confirmed our hypothesis that some individuals perceived that their condition(s) had no impact or relevance to some items. For most of the items (30 of 36), over 70% of imputed values were imputed as “easily” or “very easily”. For the remaining items (5 items from the Internal domain and 1 item from the Social Interactions domain), the imputed values were more evenly distributed, imaging original data distribution. Five distinct data sets were generated using different random number seeds to assess the impact of randomness in the imputation process, and results were compared between them. As model parameters and fit statistics were nearly identical, one imputed data file was randomly chosen to report study results.

### Assessment of structural validity

Structural validity, an aspect of construct validity, is the degree to which indicators and scores of an instrument reflect the construct to be measured [37]. It was assessed using IRT multidimensional and domain-specific graded response models estimated using the R “mirt” package [38]. The primary model, estimated using Monte Carlo Markov Chains (Metropolis-Hastings algorithm), was a seven-domain, correlated-factor model with each item having stochastically independent error terms and loading only on its respective domain. Domain specific IRT models were also estimated with limited-information maximum-likelihood to assess the structural validity of each domain. We also estimated a 7-domain correlated-factor CFA model using diagonally weighted least squares and pairwise polychoric correlations using the R “Lavaan” package [39]. This model is substantially similar to the multidimentional IRT graded response model and provides additional ways to assess model specification and item performance. In particular, modification indices were computed from the CFA model to provide evidence of any cross-loadings of items between domains (indicative of poor discriminant validity) or correlated errors (which would violate the independence assumption of IRT models) [40]. For all models, the “strongly disagree” and “disagree” response options for the Disease Controlling domain items were collapsed due to small cell sizes.

Using these models, structural validity was assessed by examining the fit of individual items, the fit of individual domains, and overall fit and validity and assumptions of the full multidimensional model. The fit of the 36 items in the English version (Swedish translation) of the PRISM-CC to the seven PRISM-CC domains was assessed based on IRT item discrimination parameters, standardized factor loadings, item-category response curves, infit and outfit mean square fit statistics, and item information. IRT item discrimination parameters in the range 1.35–1.69 were considered high, and those ≥ 1.70 were considered very high [41]. Standardized factor loadings of 0.60–0.74 were interpreted as high, and values ≥0.75 were interpreted as very high [42]. Infit and outfit statistics was considered acceptable if it was in the range of 0.5–1.5 [43]. Item-category curves and item information were used to assess discrimination of response categories for items and the contribution of each item to the measure. CFA modification indices were used to assess the magnitude of potential cross-loadings to other domains, and the extent of correlated errors between items in each domain. One item was found to have poor discriminant validity. A conceptually similar and better performing item from the translated Swedish item bank was used as replacement and used for further assessment of the structural validity.

Structural validity was further assessed based on goodness-of-fit statistics for the multi-dimensional and domain-specific IRT graded response models. Limited-information M2* fit statistics (or for individual domains with which had fewer degrees of freedom, hybrid C2 statistics) were used: Root Mean Square Error of Approximation (RMSEA), Standardized Root Mean Squared Residual (SRMSR), Tucker Lewis Index (TLI) and Comparative Fit Index (CFI) [44]. Commonly used cut-offs indicative of a good fit were used (SRMSR < 0.05, RMSEA < 0.06, and TLI and CFI > 0.95) [45]. Cronbach’s alpha and marginal reliability were also estimated for individual domains. Finally, residuals between observed and model-predicted inter-item correlations from the CFA model were examined to identify any local areas of poor fit. Residual correlations with an absolute value of >0.10 were used to indicate local areas of poor fit [46].

The population-based sample for this study was heavily skewed towards persons reporting low impact of their condition(s) on everyday life (Table 1) and corresponding low percentages reporting difficulty with managing many of the PRISM-CC items. Accordingly, a sensitivity analysis was conducted to assess the structural validity of the PRISM-CC among the 57% of subjects reporting the three highest levels of impact of their condition(s) on daily life (“moderately”, “quite a bit”, or “extremely”). Poor structural validity in this subsample would be a concern, as the need to assess self-management difficulty is most salient for those most likely to require self-management support and interventions.

### Test-retest

Test-retest reliability was assessed separately for each domain based on scores estimated with maximum a-posteriori method and then scaled to represent the range of the 6-level adjectival response scale [1–6]. Both the intra-class correlation coefficient (ICC) and Bland-Altman plots were used. ICC estimates and 95% confidence intervals were calculated using Stata 17 [34] based on a single-measurement, absolute-agreement, 2-way mixed-effects model between baseline and the 3-week follow-up [47, 48]. As suggested by Koo and Li [48], ICC values <0.50 were considered poor, values between 0.50 and 0.75 were considered moderate, values between 0.75 and 0.90 were considered good, and values >0.90 were considered excellent. Major limitations of the ICC for assessing reliability are that it depends on variance of the latent trait in the sample, as well as measurement error, and that it does not reveal differences in reliability by levels of the latent trait. Accordingly, Bland-Altman plots were used to visually assess test-retest agreement. The difference in score between the measurements was plotted against the mean score at timepoint one and two, with limits of agreement showing the interval within which about 95% of the differences between the two measurements should lie [49, 50].

## Results

### Participant characteristics

Of the 1117 individuals invited to participate; 542 (48.5%) met the inclusion criteria of having at least one long-term health condition. Of those, twelve were excluded because they did not answer the PRISM-CC section of the survey, and 14 because of missing or NA responses > 50%. This left 516 participants for inclusion in the psychometric analysis, which is a sufficient sample size to assess model fit [51]. The included sample (Table 2) reported varying chronic conditions and 77.5% (n = 400) had multimorbidity. Gender was equally distributed between females 256 (49.6%) and males 249 (48.3%). Because of the recruitment strategy via the HAI, all were between 72 and 73 years of age. Seventy-three participants (14.2%) reported no perceived impact of their condition(s) on life.

**Table 2:** Demographic and clinical characteristics of included participants

Characteristic	Total sample (n = 516)	Test-retest sample (n = 58)
	N (%)	N (%)
**Gender**		
Female	256 (49.6)	28 (48.3)
Male	249 (48.3)	30 (51.7)
Missing	11 (2.1)	0
**Living situation**		
Live alone	114 (22.1)	17 (29.3)
Shared household	390 (75.6)	40 (69.0)
Missing	12 (2.3)	1 (1.7)
**Civil status**		
Married/partner	386 (74.8)	40 (69.0)
Living apart	12 (2.3)	2 (3.5)
Widow/widower	29 (5.6)	5 (8.6)
Single	74 (14.3)	10 (17.2)
Missing	15 (2.9)	1 (1.7)
**Highest level of education completed**		
Incomplete elementary school	3 (0.6)	1 (1.7)
Elementary school	102 (19.8)	13 (22.4)
High school	158 (30.6)	17 (29.3)
Graduate degree	235 (45.5)	26 (44.8)
Missing	18 (3.5)	1 (1.7)
Conditions*		
Cardiovascular disease	402 (77.9)	49 (84.5)
Neurological condition	54 (10.5)	7 (12.1)
Metabolic disease	132 (25.6)	12 (20.7)
Muscular skeletal disease	250 (48.5)	34 (58.6)
Respiratory disease	97 (18.8)	18 (31.0)
Gastro or bowel disease	77 (14.9)	10 (17.2)
Urinary or kidney disease	135 (26.7)	15 (25.9)
Skin condition	91 (17.6)	15 (25.9)
Disability/injury after accident	58 (11.2)	17 (29.3)
Other	113 (21.9)	8 (13.8)
**Count of chronic conditions**		
1	116 (22.5)	8 (13.8)
2	144 (27.9)	11 (19.0)
3	127 (24.6)	17 (29.3)
4 +	129 (25.0)	22 (37.9)
**Perceived impact of disease(s) on life**		
Extremely	24 (4.7)	3 (5.2)
Quite a bit	92 (17.8)	10 (17.2)
Moderately	176 (34.1)	18 (31.0)
A little bit	139 (26.9)	15 (25.9)
Not at all	73 (14.2)	11 (19.0)
I don´t know	4 (0.8)	1 (1.7)
Missing	8 (1.6)	0

^*^ = participants may have more than one condition

### Item and domain characteristics

For item and domain characteristics, see Table 1. Missing values due to non-response were low for all 36 items, ranging between 0.2 to 1.7%. Not Applicable responses ranged from 0.8 to 31.2%, with 11 items having NA responses > 10%. As expected, most imputed values for NA responses were at the higher end of the response scale (“easily” to “very easily”). Sensitivity analyses with NA responses coded as missing and not imputed minimally changed estimates of item parameters or model fit by domain.

### Structural validity and internal consistency

All 36 items from the English version of the PRISM-CC had high measurement quality. However, CFA modification indices revealed that one translated item from the Healthy Behaviours domain (“I create time in my day to be active (walk to work, do housework, yard work or other daily activities”)) cross-loaded with the Activities domain and thus had poor discriminant validity. This item was replaced with a conceptually similar and more generally formulated item (Hea5S– “I maintain healthy behaviours even when I have a lot to do”), which performed well and did not have the same cross-loading problem. With this one change, all IRT-graded response discrimination parameters and their corresponding standardised loadings were very high (>1.7 and > 0.7, respectively). Infit and outfit statistics for most items were close to 1.0, and all were in the range of 0.5 to 1.5 (Table 3). Item information curves showed high item information throughout, especially at the response scale’s lower end (perceived difficulty). Item response curves (Supplementary File 3) showed all items and their polytomous responses discriminated across levels of the domains; however, the response curves for some items (provided in supplemental material), especially in the Resource domain, had a high degree of overlap between the 2^nd^ and 3^rd^ lowest categories (“with extreme difficulty” and “with much difficulty”), suggesting that subjects may experience difficulty differentiating between these two response categories. This may also reflect imprecise estimation due to low frequencies for these responses in many items.

**Table 3:** Item parameters and fit statistics for the multidimensional graded response model

Domain and item	Discrim	Std. loading	Item diff	Outfit	Infit
**Resource**					
Res1	3.36	0.89	−2.07	0.79	0.96
Res2	3.20	0.88	−2.05	0.69	0.95
Res3	3.29	0.89	−2.02	0.58	0.88
Res4	2.83	0.86	−2.12	0.67	1.09
**Process**					
Pro1	2.79	0.85	−1.84	0.78	0.89
Pro2	4.42	0.93	−2.20	0.67	0.96
Pro3	4.10	0.92	−1.67	0.75	0.88
Pro4	3.57	0.90	−2.05	0.86	1.03
Pro5	2.70	0.85	−2.09	0.89	0.93
**Internal**					
Int1	2.61	0.84	−2.05	0.85	0.92
Int2	2.46	0.82	−1.81	0.93	0.98
Int3	3.46	0.90	−1.94	0.89	1.02
Int4	3.22	0.88	−1.86	0.82	0.99
Int5	2.57	0.83	−2.05	0.98	0.99
Int6	2.31	0.81	−1.77	0.91	0.94
Int7	2.25	0.80	−1.49	0.94	0.99
Int8	1.91	0.75	−1.74	0.95	0.98
**Activities**					
Act1	2.29	0.80	−2.09	0.86	0.90
Act2	2.31	0.81	−2.43	0.86	0.97
Act3	3.32	0.89	−2.34	0.70	0.91
Act4	4.05	0.92	−2.00	0.59	0.80
Act5	2.90	0.86	−2.19	0.96	1.07
**Social Interaction**					
Soc1	2.34	0.81	−2.17	0.84	0.96
Soc2	2.44	0.82	−2.23	0.78	0.84
Soc3	2.23	0.79	−1.53	0.89	0.90
Soc4	2.13	0.78	−2.82	0.77	0.94
Soc5	2.55	0.83	−2.22	0.82	0.99
**Healthy Behaviours**					
Hea1	3.54	0.90	−1.98	0.75	1.01
Hea2	2.07	0.77	−2.13	0.85	0.92
Hea3	1.80	0.73	−2.60	0.90	0.98
Hea4	1.75	0.72	−1.73	0.95	1.01
Hea5S	3.44	0.90	−1.70	0.78	0.86
**Disease Controlling**					
Dis1	2.75	0.85	−2.17	0.65	0.85
Dis2	2.66	0.84	−1.89	0.69	0.89
Dis3	3.16	0.88	−1.81	0.58	0.81
Dis4	2.62	0.84	−1.91	0.65	0.91

Note: Discrim = Discrimination parameter, Item difficulty is generalized item difficulty based on the item response function from the R mirt package

With the substituted item in the Healthy Behaviours domain, the full and domain-specific models had good fit to the data. For the full model, correlations between domains ranged between 0.440–0.833, and fit statistics demonstrated good fit (TLI = 0.945, CFI = 0.952, SRSMR = 0.050, RMSEA = 0.034, 95% CI: 0.029–0.038). The CFI, TLI, and SRMSR fit statistics for domain-specific models showed good fit; however, the RMSEA was higher than the recommended cut-off of 0.06 for all but the Disease Controlling domain (Table 4). Cronbach’s alpha was > 0.8 for all domains and > 0.9 for two domains. There was minimal evidence of local areas of poor fit. In the CFA residual correlation matrix, only two (of 36) residual correlations exceeded the criteria (0.167 between int7 and int8, and 0.138 between soc3 and soc4). Modification indices revealed this was due to correlated errors between the items; however, they were not substantial enough to warrant dropping or replacing items.

**Table 4:** Estimated Cronbach’s alpha, fit indices and marginal reliability by PRISM-CC domain

	Resource	Process	Internal	Activities	Social Interaction	Healthy Behaviours	Disease Controlling
Cronbach’s alpha	0.86	0.91	0.91	0.88	0.84	0.84	0.84
Graded response models							
MR	0.78	0.85	0.91	0.82	0.82	0.85	0.73
CFI	0.99	0.99	0.99	0.99	0.99	0.99	1.00
TLI	0.97	0.99	0.98	0.99	0.98	0.97	1.00
C2 RMSEA	0.10	0.08	0.07	0.07	0.07	0.09	0.00
95% LCI	0.05	0.05	0.05	0.04	0.04	0.05	0.00
95% UCI	0.16	0.11	0.09	0.11	0.11	0.12	0.08
SRMSR	0.04	0.04	0.04	0.03	0.04	0.05	0.02

Note: MR = Marginal Reliability, CFI = Comparative Fit Index, TLI = Tucker–Lewis index, RMSEA = root mean square error of approximation, SRMSR = standardized root mean squared residuals, LCI = lower confidence interval, UCI = upper confidence interval

Sensitivity analyses were conducted, restricting the sample to the 56.6% of subjects (N = 292) reporting at least moderate impact of their condition(s) on everyday life. Fit of the full model in the restricted sample was only slightly diminished relative to the full sample (TLI = 0.925, CFI = 0.934, SRMSR = 0.061, RMSEA = 0.038. CI: 0.031. - 0.045).

### Test-retest reliability

Nighty-one of the 100 invited participants returned the retest survey. Thirty-three participants were excluded because the response time exceeded the 3-week test-retest period or because the NA – or missing responses exceeded > 50%. The remaining fifty-eight participants had characteristics similar to the total sample (Table 2). ICCs were good for all domains except for the Disease Controlling domain that showed a moderate ICC (Table 5).

**Table 5:** Test-retest by domain using Intraclass Correlation (ICC) 2-way mixed-methods model with absolute agreement, single measurement and Bland-Altman

	ICC			Bland-Altman					
Domain	ICC	95% CI Lower Bound	95% CI Upper Bound	Bias	95% CI	Lower LoA	95% CI	Upper LoA	95% CI
Resource	0.76	0.63	0.85	0.10	-0.20, 0.01	-0.88	-1.06, -0.70	0.69	0.51, 0.87
Process	0.76	0.61	0.85	0.13	-0.24, -0.02	-0.93	-1.11, -0.75	0.67	0.48, 0.85
Internal	0.89	0.82	0.93	0.04	-0.13, 0.04	-0.68	-0.83, -0.53	0.59	0.45, 0.74
Activities	0.86	0.77	0.91	0.04	-0.12, 0.05	-0.67	-0.82, -0.53	0.59	0.45, 0.74
Social Interaction	0.79	0.67	0.87	0.04	-0.13, 0.05	-0.71	-0.86, -0.55	0.63	0.48, 0.78
Healthy Behaviours	0.82	0.71	0.89	0.10	-0.19, -0.01	-0.77	-0.93, -0.62	0.57	0.42, 0.73
Disease Controlling	0.53	0.31	0.69	0.02	-0.07, 0.12	-0.67	-0.84, -0.51	0.72	0.56, 0.88

ICC = Intraclass correlation, Bias = mean difference, LoA = Limit of Agreement

**Fig. 2:**
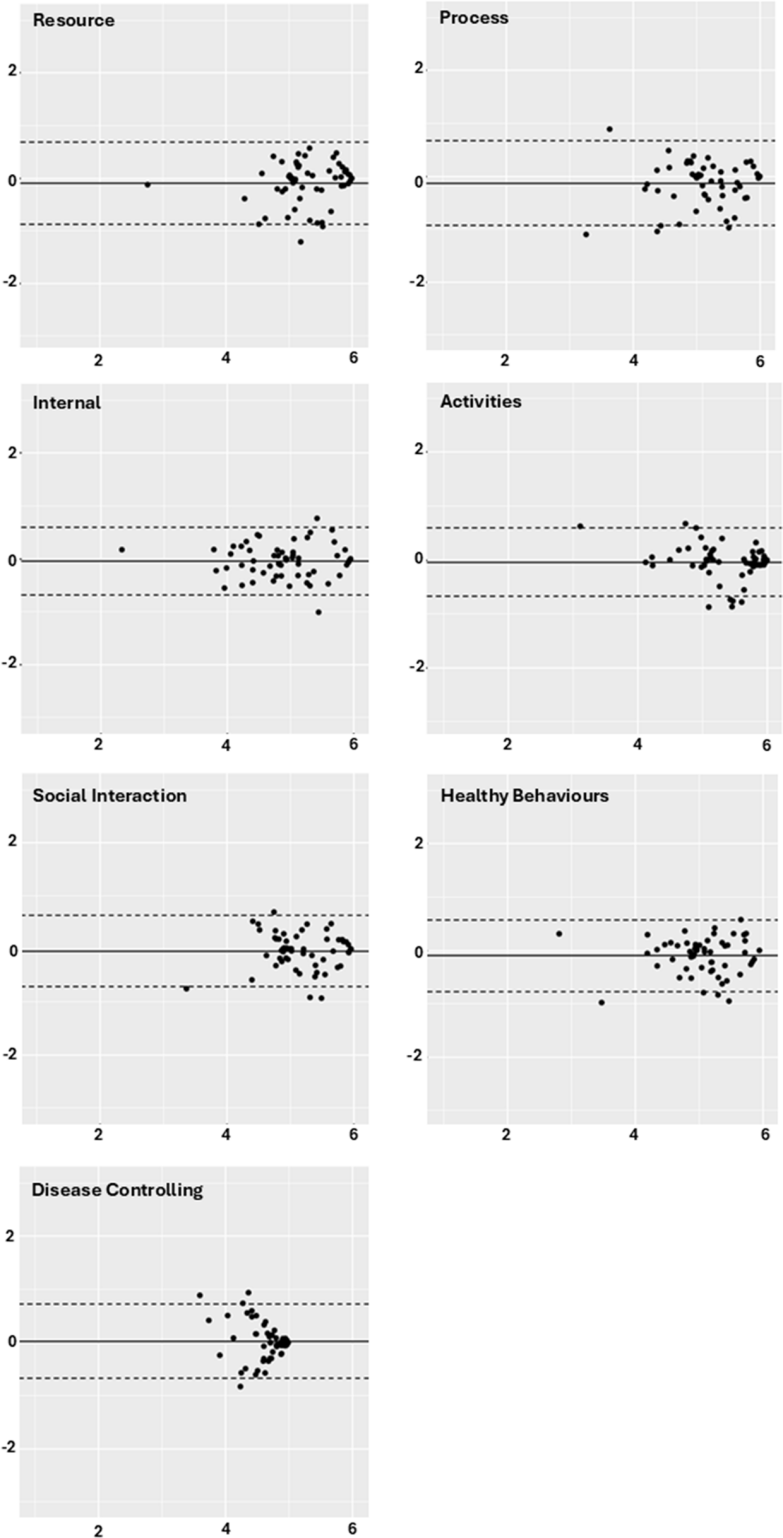
Bland-Altman plots comparing timepoint one and two for the seven PRISM–CC domains. The plot displays the mean of the two timepoints on the x–axis and the difference in scores on the y–axis. The solid line represents the mean difference, while the dotted lines indicate the 95% limits of agreement

Bland-Altman plots showed minimal bias, with the limits of agreement within 0.50–0.75 for all domains, corresponding to an average difference of less than one adjectival unit on the 6-level response scale. The Bland-Altman plots also highlight the relatively low level of sample variation among the test-retest sample, especially in the Disease Controlling domain. This contributes to lower ICCs since the denominator of the ICC is the total variation in measurement (Fig. 2). Thus, the lower ICC for the Disease Controlling domain is due to low variation in the sample, not low levels of test-retest agreement.

## Discussion

In this study, we investigated the structural validity and test-retest reliability of a Swedish version of the PRISM-CC in a sample of seventy-year-olds with long-term health conditions. Consistent with the English version of the PRISM-CC [27], this study found evidence of structural validity of the Swedish version, with good item quality, and good fit to the full factor and domain-specific IRT models. There are, however, some caveats to this conclusion. First, for some items, response characteristic curves suggest that respondents may have difficulty distinguishing between the 2nd and 3rd lowest response categories, but this was not observed in the validation of the English version of the PRISM-CC and may be the result of low response frequencies for these response categories in the Swedish data. A second caveat is that the estimated upper confidence intervals for the RMSEA in many of the CFA and IRT domain specific models were above the widely used cut-off for good fit (0.06). We do not believe this detracts from the evidence of strong structural validity, however, since simulation studies have shown that in the presence of standardized factor loading greater than 0.70, which was found for all items in this study, RMSEA values higher than 0.06 can indicate good fit [42]. The English version of the PRISM-CC showed similar patterns regarding high factor loadings and RMSEA-values over the 0.06 cut-off [27]. The third caveat was the poor discriminant validity of one item in the Healthy Behaviours domain. A more generally formulated item in the English version performed better than items measuring specific behaviours, prompting the substitution of one item in the Swedish version. Future refinement of the English version could consider a similar substitution. Although cross-loadings of items between domains were low, correlations between domains were high. This was expected, as the TEDSS framework emphasises the interrelatedness of domains [14, 15]. While Cronbach’s alpha was high for all domains, this is not a result of item redundancy. During the item selection process, items were selected to reflect the content of the domains and items with similar meaning and content were not included. Moreover, correlated errors between items, identified using modification indices in the CFA analyses, were used to identify and choose between items with correlated errors (which are generally high between redundant items).

A key limitation of this study was the relatively healthy sample, with only about half reporting significant impact of their health condition(s) on daily life. Item responses skewed towards little or no difficulty, with a substantial number of NA responses that imputation and analyses suggests resulted from lack of relevance of some items to respondents’ health conditions. This limits the strength of evidence about the validity of the Swedish version of the PRISM-CC for persons with greater self-management difficulty. However, sensitivity analyses showed that excluding those with little or no perceived impact of their condition(s) on daily life did not appreciably affect model fit. Moreover, the results of this study should be considered in the context of strong evidence for structural and construct validity in the English version of the PRISM-CC [27]. Validation of the English version employed a much larger sample, which was skewed towards high impact of health conditions on daily life. Given the joint English-Swedish development of the PRISM-CC, and the matching evidence for structural validity in both the Swedish and English data, confidence in the robustness of our results to sample composition is enhanced.

The TEDSS framework that the PRISM-CC is based on was originally developed by interviewing individuals with neurological conditions [14]. This could be seen as a limitation when developing a measure for generic use. However, about 40% of the participants in the TEDSS study had comorbid non-neurological conditions and the framework has been presented for patient associations representing different diseases, and health-care providers working in various specialities. When developing the PRISM-CC, the more disease-specific items were removed during the item selection to ensure its suitability for generic use and cognitive interviews ensured relevance for people with various diseases.

Another limitation of this study concerns test-retest reliability. An assumption for test-retest reliability is that there has been no change in the measured construct between timepoints one and two. However, there is always a possibility of disease or circumstantial change between responses [52]. This study had a low response rate within the time frame. After removing individuals with delayed response and those with more than 50% missing responses in one or more domains, only 58 participants were eligible for inclusion. Test-retest showed good ICCs for all domains except for the Disease Controlling domain. However, the Bland-Altman plot reveals that the limits of agreement were similar to other domains. Therefore, lower ICC in the Disease Controlling domain resulted from the small variance within the sample relative to the variance within subjects. This is a well-documented limitation of the ICC [49, 50]. The test-retest reliability of the PRISM-CC should be further investigated in a clinical sample with a higher level of self-management difficulty and with greater impact of their conditions on everyday life.

The development of a patient-reported outcome measure is a stepwise process. In the next stage of the PRISM-CC development, Differential Item Functioning (DIF) should be conducted to provide insight into how the PRISM-CC performs for specific groups and among people with different diagnoses. As well, the Swedish version of the PRISM-CC should be tested in clinical settings, especially if further validation procedures are performed simultaneously. Testing in clinical samples with participants that experience a more significant impact of their disease(s) on daily life would render more information on how the instrument performs among people with greater self-management difficulty.

## Conclusion

The Swedish version of the PRISM-CC showed good structural validity and test-retest reliability in a sample of relatively healthy seventy-year-olds with long-term health condition(s). The results are promising and supports the assumption that the PRISM-CC is a viable instrument for assessing patient-reported self-management difficulty for people with significant impact of their condition(s) on daily life. Because it is generic and relatively short, the PRISM-CC is suitable for use in primary health care and for people with multi-morbidity. However, further validation in a population more significantly affected by their condition in daily life is needed before it can be integrated into routine care.

## Electronic supplementary material

Below is the link to the electronic supplementary material.

Supplementary Material 1

Supplementary Material 2

Supplementary Material 3

## Data Availability

The dataset generated and analysed during the current study is not publicly available because participant consent included restrictions on the use of the data due to patients’ privacy concerns. Limited availability is possible. Researchers wishing information may contact ÅA.

## Abbreviations

TEDSS: Taxonomy of Everyday Self-management Strategies
heiQ: Health Education Impact Questionnaire
PRISM-CC: Patient Reported Inventory of Self-Management of Chronic Conditions
COSMIN: Consensus-based Standards for the selection of health Measurement Instruments
PROMIS®: Patient Reported Outcomes Measurement Information System
HAI: Healthy Aging Initiative
CFA: Confirmatory factor analysis
IRT: Item response theory
NA: Not applicable
PRO: Patient-Reported Outcome
RMSEA: Root Mean Square Error of Approximation
SRMSR: Standardized Root Mean Squared Residual
TLI: Tucker Lewis Index
CFI: Comparative Fit Index
ICC: Intra-class correlation coefficient
