# Supplementary material for: Structural validity and test-retest reliability of the Patient Reported Inventory of Self-Management of Chronic Conditions (PRISM-CC) in a Swedish population of seventy-year-olds with long-term health conditions
Source: J Patient Rep Outcomes. 2025 May 28;9:59. doi: 10.1186/s41687-025-00892-3 (PMC12119446; doi:10.1186/s41687-025-00892-3)
Supplement: Supplementary file 1 — Supplementary Material 1 [file 41687_2025_892_MOESM1_ESM.pptx]

## Slide 1
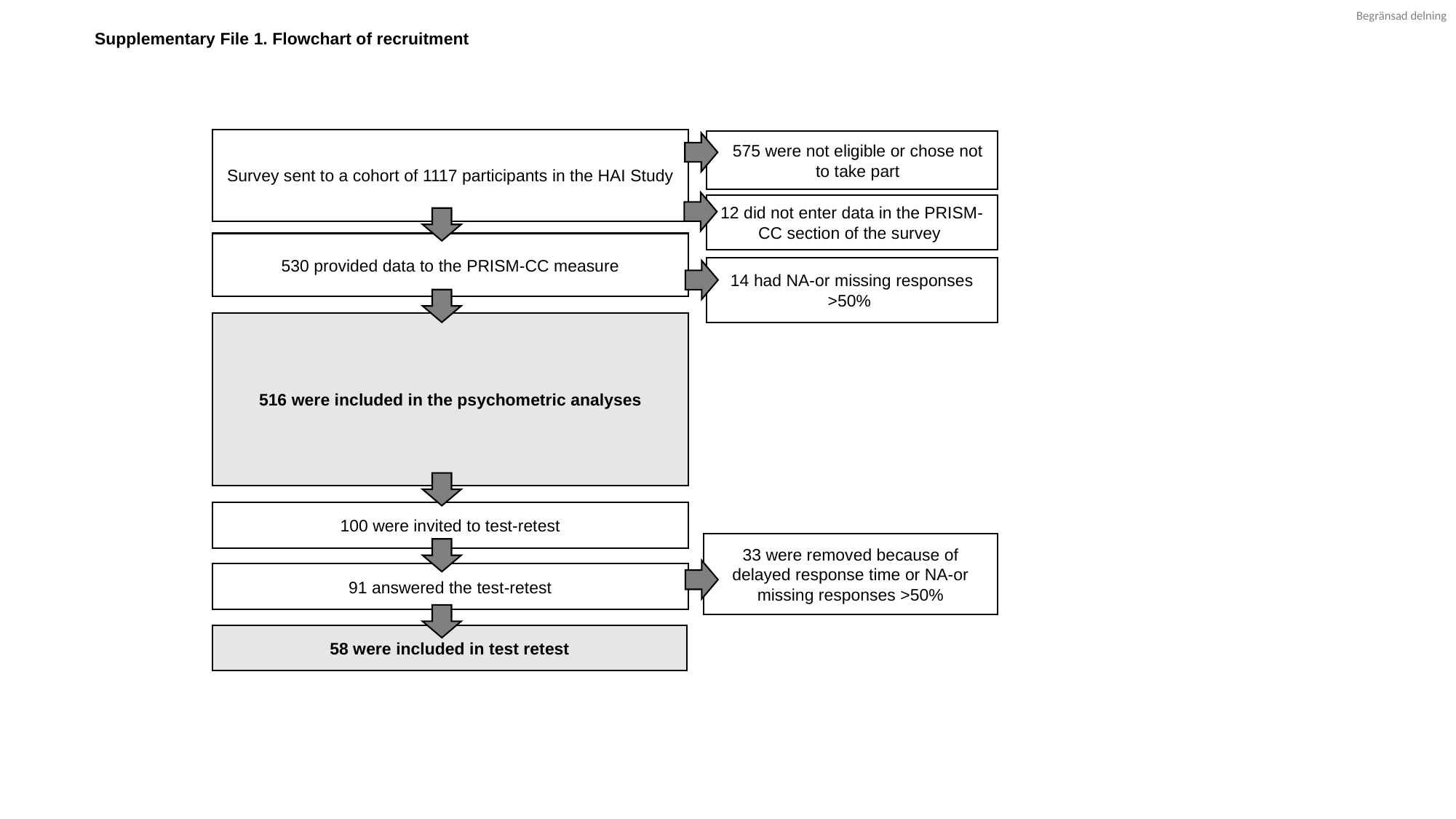

Supplementary File 1. Flowchart of recruitment
Survey sent to a cohort of 1117 participants in the HAI Study
575 were not eligible or chose not to take part
12 did not enter data in the PRISM-CC section of the survey
530 provided data to the PRISM-CC measure
14 had NA-or missing responses >50%
516 were included in the psychometric analyses
100 were invited to test-retest
33 were removed because of delayed response time or NA-or missing responses >50%
91 answered the test-retest
58 were included in test retest
