# Supplementary material for: Structural validity and test-retest reliability of the Patient Reported Inventory of Self-Management of Chronic Conditions (PRISM-CC) in a Swedish population of seventy-year-olds with long-term health conditions
Source: J Patient Rep Outcomes. 2025 May 28;9:59. doi: 10.1186/s41687-025-00892-3 (PMC12119446; doi:10.1186/s41687-025-00892-3)
Supplement: Supplementary file 2 — Supplementary Material 2 [file 41687_2025_892_MOESM2_ESM.docx]

| **Supplementary File 2** | |  |  |  |  |  |  |
| --- | --- | --- | --- | --- | --- | --- | --- |
| Correlation between items with a NA response >10% and 1) participants who reported that their condition had no impact on life and 2) participants with only one disease | | | | | |  |  |
|  |  |  | **1** |  | **2** |  |  |
| **Item** |  | **NA responses (%)** | **Fisher exact** |  | **Fisher exact** |  |  |
| **Res1** | When I have appointments with my healthcare providers, I tell them what I want or need. | 62 (12.0) | 0.031 | * | 0.049 | * |  |
| **Res2** | I talk to my healthcare provider(s) about my condition(s). | 100 (19.4) | 0.002 | * | 0.014 | * |  |
| **Res3** | I arrange appointments with my health care provider(s). | 52 (10.1) | 0.005 | * | 0.859 |  |  |
| **Res4** | When I need to, I find people to help me understand information I receive about my condition(s). | 161 (31.2) | 0.006 | * | 0.008 | * |  |
| **Pro1** | I identify what information I can trust. | 75 (14.5) | 0.370 |  | 0.015 | * |  |
| **Pro4** | I try different things to find out what works best for me. | 75 (14.5) | 0.010 | * | 0.035 | * |  |
| **Int5** | I deal with frustration caused by my health situation. | 76 (14.7) | 0.000 | * | 0.000 | * |  |
| **Act1** | I organize things in my home to make my life easier. | 56 (10.9) | 0.419 |  | 0.230 |  |  |
| **Act2** | I plan ahead before going somewhere to be sure I can manage my health condition(s). | 99 (19.2) | 0.025 | * | 0.030 | * |  |
| **Soc3** | I clearly express my needs to others. | 59 (11.4) | 0.000 | * | 0.004 | * |  |
| **Soc5** | When problems with my health arise, I stay in touch with people who are important to me. | 56 (10.9) | 0.000 | * | 0.000 | * |  |
| * Significant p-value <0.05 | |  |  |  |  |  |  |
