# Supplementary material for: Structural validity and test-retest reliability of the Patient Reported Inventory of Self-Management of Chronic Conditions (PRISM-CC) in a Swedish population of seventy-year-olds with long-term health conditions
Source: J Patient Rep Outcomes. 2025 May 28;9:59. doi: 10.1186/s41687-025-00892-3 (PMC12119446; doi:10.1186/s41687-025-00892-3)

**Supplementary File 3. Item response curves of the Swedish version of the PRISM-CC**

Response option 1 -Difficulty

Response option 2

Response option 3

Response option 4

Response option 5

Response option 6 -Ease

**Activities**

**Act1**

I organize things in my home to make my life easier.


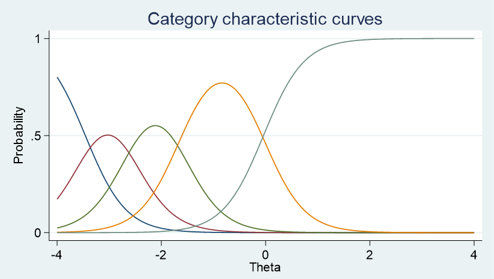


**Act2**

I plan ahead before going somewhere to be sure I can manage my health condition(s).

**
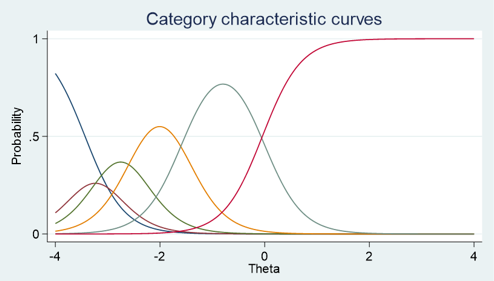
**

**Act3**

I plan my time so I can get things done.


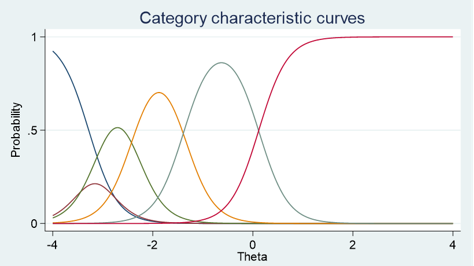


**Act4**

I manage my health condition(s) so that I can do things I enjoy.

**
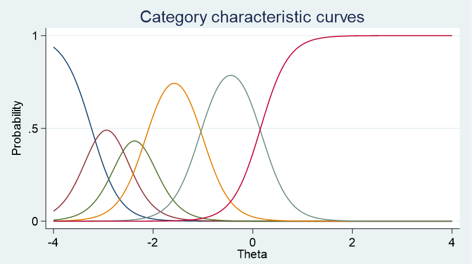
**

**Act5**

I make time to do things I enjoy.


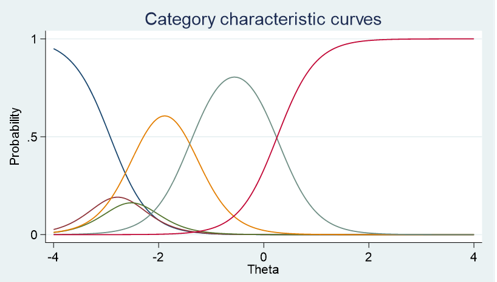


**Social Interaction**

**Soc1**

I prioritize social interactions that I enjoy.

**
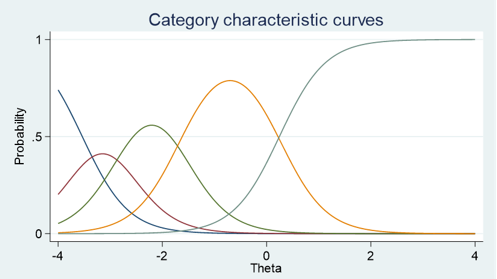
**

**Soc2**

I can explain my symptoms so family and friends can understand them.


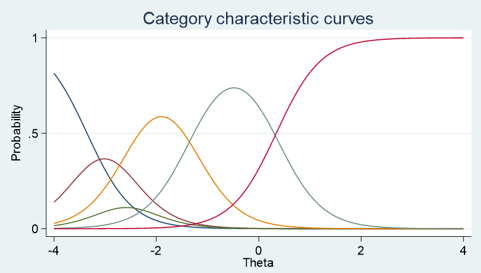


**Soc3**

I clearly express my needs to others.

**
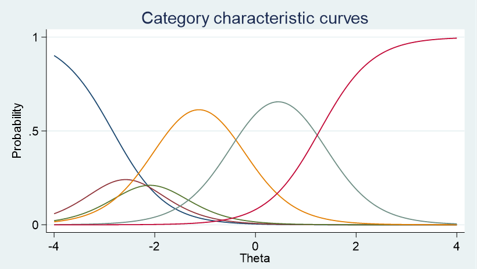
**

**Soc4**

I devote time and attention to those who are dear to me.


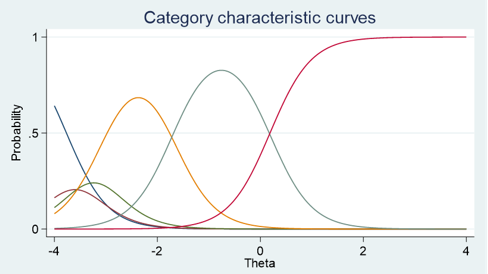


**Soc5**

When problems with my health arise, I stay in touch with people who are important to me.

**
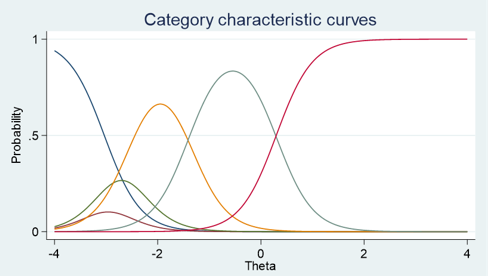
**

**Internal**

**Int1**

I set realistic expectations for myself.

**
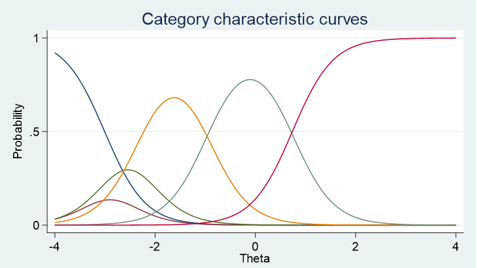
**

**Int2**

I accept the things I cannot change.


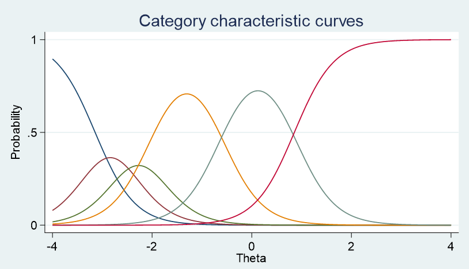


**Int3**

I manage my emotions and reactions.

**
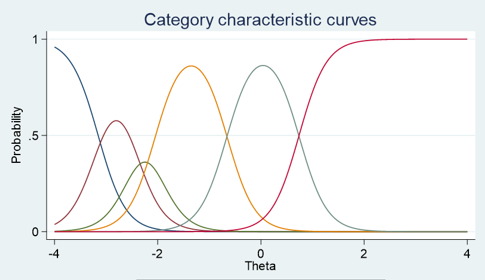
**

**Int4**

I have and use ways to recover after a bad day.


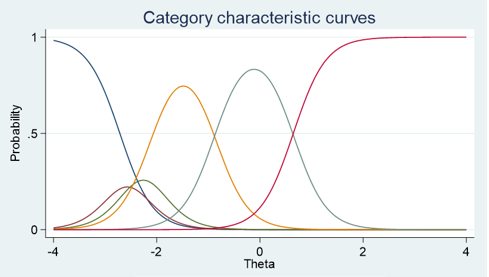


**Int5**

I deal with frustration caused by my health situation.


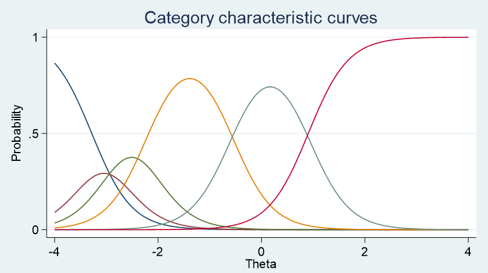


**Int6**

I manage my stress.

**
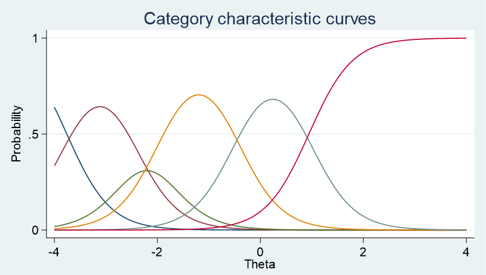
**

**Int7**

I focus on the positives.

**
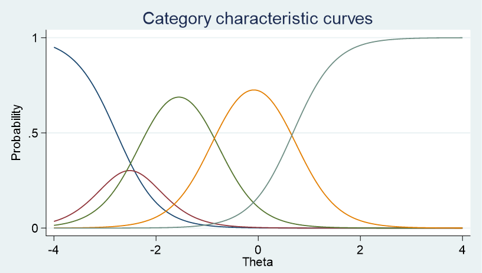
**

**Int8**

I forgive myself when I make a mistake.


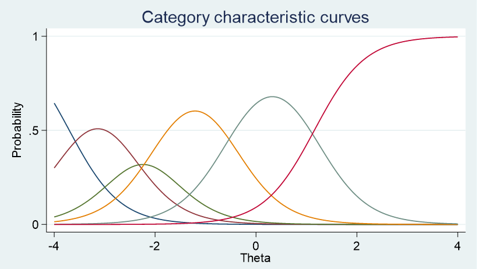


**Healthy Behaviours**

**Hea1**

I maintain healthy lifestyle behaviours that I know are important for my health.

**
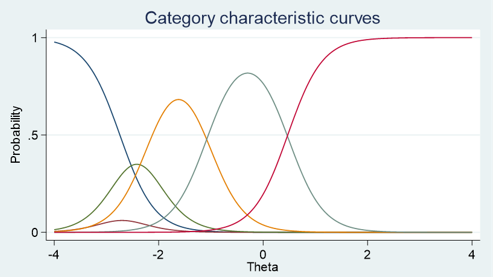
**

**Hea2**

I make healthy food choices.

**
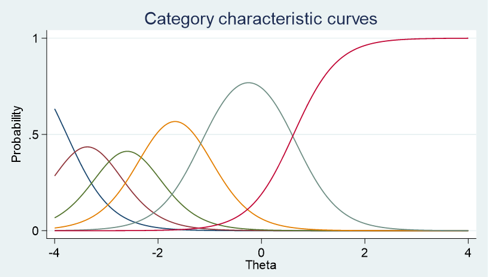
**

**Hea3**

I find ways to train my brain to keep mentally fit.


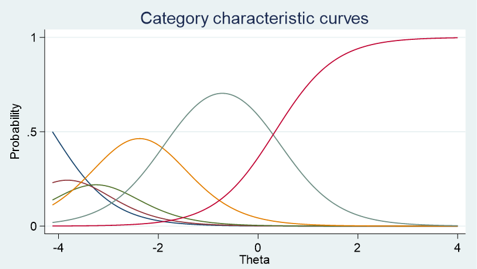


**Hea4**

I create healthy sleeping habits.

**
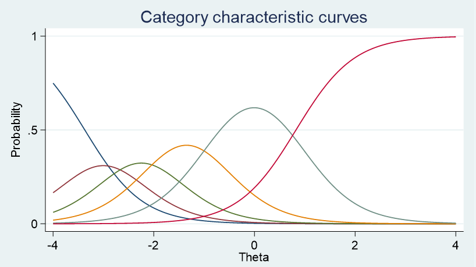
**

**Hea5S**

I maintain healthy behaviours even when I have a lot to do.


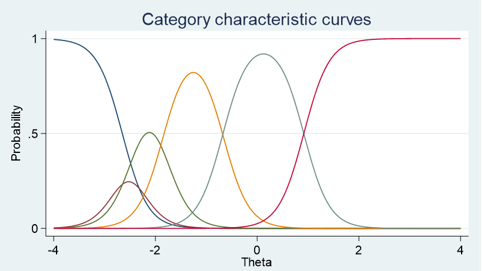


**Disease Controlling**

**Dis1**

When problems with my health arise, I understand what to do to manage my condition(s).

**
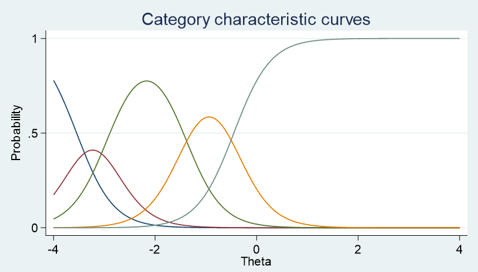
**

**Dis2**

I know what to do if I experience side-effects or other problems with my treatment or medication.

**
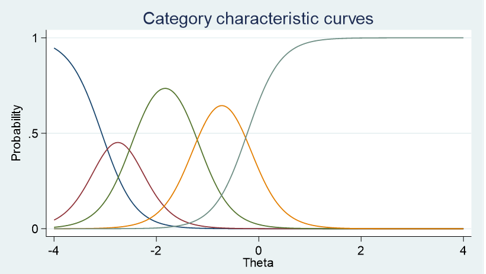
**

**Dis3**

I know which symptoms I need to act upon.

**
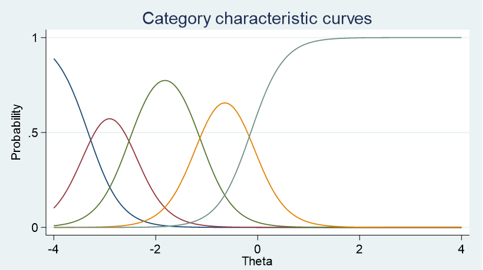
**

**Dis4**

I know what to do when my symptoms get worse.

**
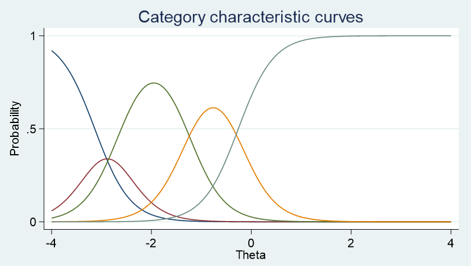
**

**Process**

**Pro1**

I identify what information I can trust.


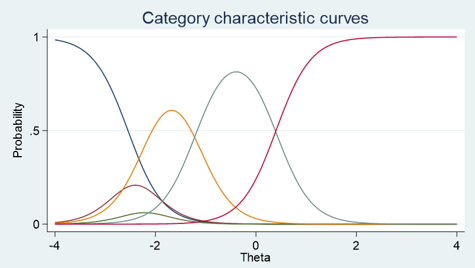


**Pro2**

I make informed decisions.

**
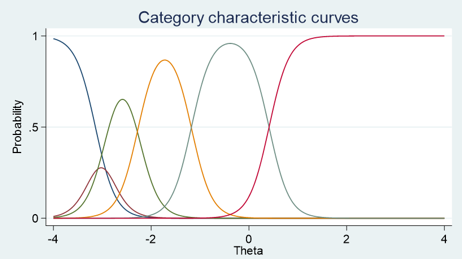
**

**Pro3**

I think about the consequences of different decisions.


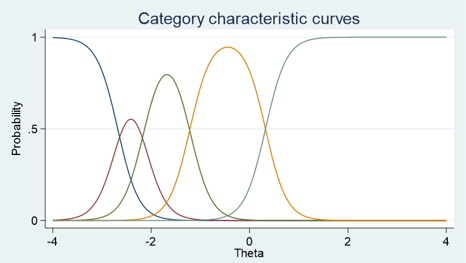


**Pro4**

I try different things to find out what works best for me.


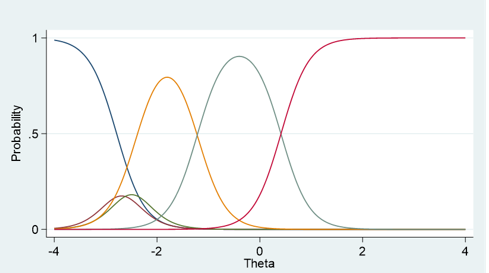


**Pro5**

I keep myself updated with new information related to my health conditions.


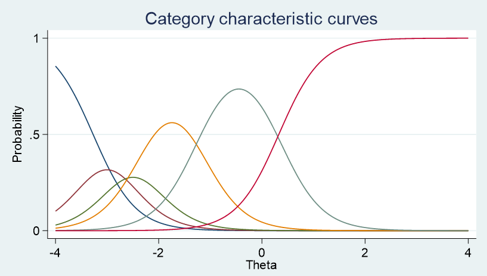


**Resource**

**Res1**

When I have appointments with my healthcare providers, I tell them what I want or need.


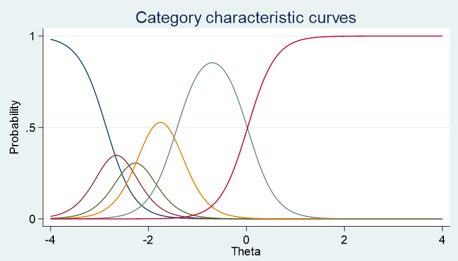


**Res2**

I talk to my healthcare provider(s) about my condition(s).

**
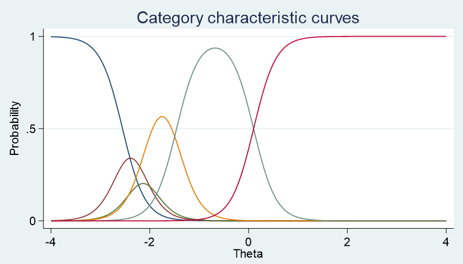
**

**Res3**

I arrange appointments with my health care provider(s).

**
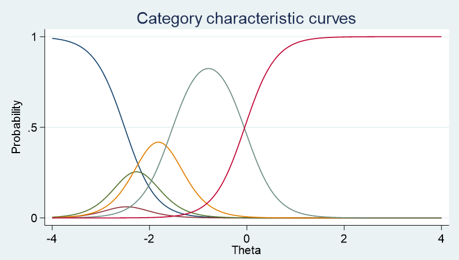
**

**Res4**

When I need to, I find people to help me understand information I receive about my condition(s).


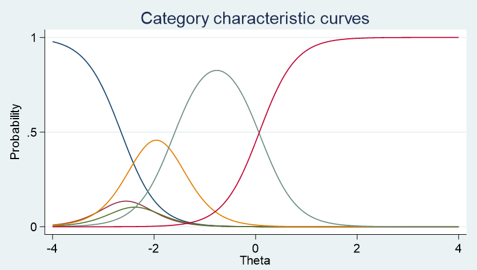

Supplement: Supplementary file 3 — Supplementary Material 3 [file 41687_2025_892_MOESM3_ESM.docx]
